# First report of northern root-knot nematode, *Meloidogyne hapla* (Chitwood, 1949) on strawberry in Turkey

**DOI:** 10.21307/jofnem-2021-002

**Published:** 2021-02-25

**Authors:** Adem Özarslandan, Dilek Dinçer, Şefika Yavuz, Ayşenur Aslan

**Affiliations:** 1 Mersin University Applied Technology and Management, School of Silifke, 339400, Silifke, Mersin, Turkey; 2 Biological Control Research Institute, Ministry of Agriculture, 01321, Yüreğir, Adana, Turkey; 3 Plant Protection Department, Faculty of Agriculture, Atatürk University, 25040, Yakutiye, Erzurum, Turkey

**Keywords:** *Meloidogine hapla*, Root-knot nematode, Strawberry

## Abstract

Strawberry is one of the most economically important crops worldwide. Several species of plant-parasitic nematodes have been reported to be pathogenic on strawberries, among them the northern root-knot nematode (*Meloidogyne hapla*), which considered to be strawberry most important nematode pest worldwide. In August 2019, strawberry growers at Silifke (Mersin, Turkey) identified nematode-like symptoms on strawberry roots and infected seedlings were brought to the nematology laboratory at of Mersin University for diagnostics. Roots were separated into small pieces and nematode extraction was performed by a modified Baermann funnel method and identified under the microscope. DNA was extracted from individual nematodes using Worm Lysis Buffer(WLB (+)). The species-specific SCAR markers (JMV1, JMV2, and JMVhapla) yielded a 440 bp band specific to *M. hapla*. The 28S rRNA gene region, obtained using the general primers D2\D3, sequence was analysed from. The analyzed sequence was 100% identicle to *M. hapla*. The gene sequences were deposited into GenBank database with accession numbers MN897751 and MN895037. Both morphological and molecular diagnostic methods confirmed that the strawberry plants collected in Silifke were infested with *M. hapla*. To our best knowledge this is the first report of plant-parasitic nematode species *M. hapla* infecting strawberry in Turkey. Currently, the adverse effect of RKN on strawberry production in the region is unknown to strawberry growers.

Strawberry is a crop with global economic significance. Strawberry is cultivated mostly in China, USA, Mexico, Egypt, Turkey, and Spain (FAOSTAT, 2019). Strawberry production is conducted in all regions in Turkey and a total production is 486 705 ton an area of 16,089 ha. In total, 65,998 ton strawberry is produced on an area of 1,650 ha in Mersin province Silifke district (TUİK, 2019). The nematode leads to significant yield losses and crop damages in several host plants due to feeding by a dense population of second-stage juvenile. Several plant-parasitic nematode species were reported to cause damages in strawberries, and the northern root-knot nematode (RKN) *Meloidogyne hapla* (Chitwood, 1949) and the northern root-lesion nematode (RLN) *Pratylenchus penetrans* (Cobb) (Filipjev and Shuurmans Stekhoven) are the most harmful nematodes worldwide (Bélair and Khanizadeh, 1994; Brown et al., 1993; Nyoike et al., 2012; Samaliev and Mohamedova, 2011). Foliar nematodes, such as Aphelenchoides fragariae (Christie, 1932; Ritzema-Bos, 1891), *Aphelenchoides ritzemabosi* (Schwartz, 1911; Steiner and Buhrer, 1932), *Aphelenchoides besseyi* (Christie, 1942), and *Ditylenchus dipsaci* (Filipev, 1936; Kühn, 1857) were reported as strawberry pests in the USA, Europe, Australia, and the former USSR (Brown et al., 1993). Needle and dagger nematodes in Longidorus and Xiphinema genera were associated with the transmission of viruses and decline in strawberry yields (Brown et al., 1993). The sting nematode, *Belonolaimus longicaudatus* (Rau, 1958), significantly restricted commercial strawberry production in Florida (Noling, 2011). Stunted plants and reduced yield were frequently associated with *M. hapla*, *P. penetrans, D. dipsaci*, and *Hemicycliophora* spp. in Spain (Bascón et al., 2012; Peña-Santiago et al., 2004; Vega et al., 2002). Nematodes open the door for soil-borne bacteria and fungi, and pests in the wounds they cause in plant root. Nematodes leads to further damages in the plant with soil-borne fungi and bacteria.

The strawberry cultivation is economically significant in Turkish agricultural industry and cultivated mainly in Mediterranean provinces such as Mersin. The production of the plant is the highest among agricultural products in Mersin province and the strawberry fields are mostly concentrated in Silifke district. In 2019, damages were observed in certain strawberry plants that resembled the root-knot nematode (*Meloidogyne* spp.) such as galling on roots and stunting. The present study was conducted to determine the type of root-knot nematode that led to small galling on the roots of strawberry plants.

## Material and method

### Nematode isolation and identification

**Figure 1:**
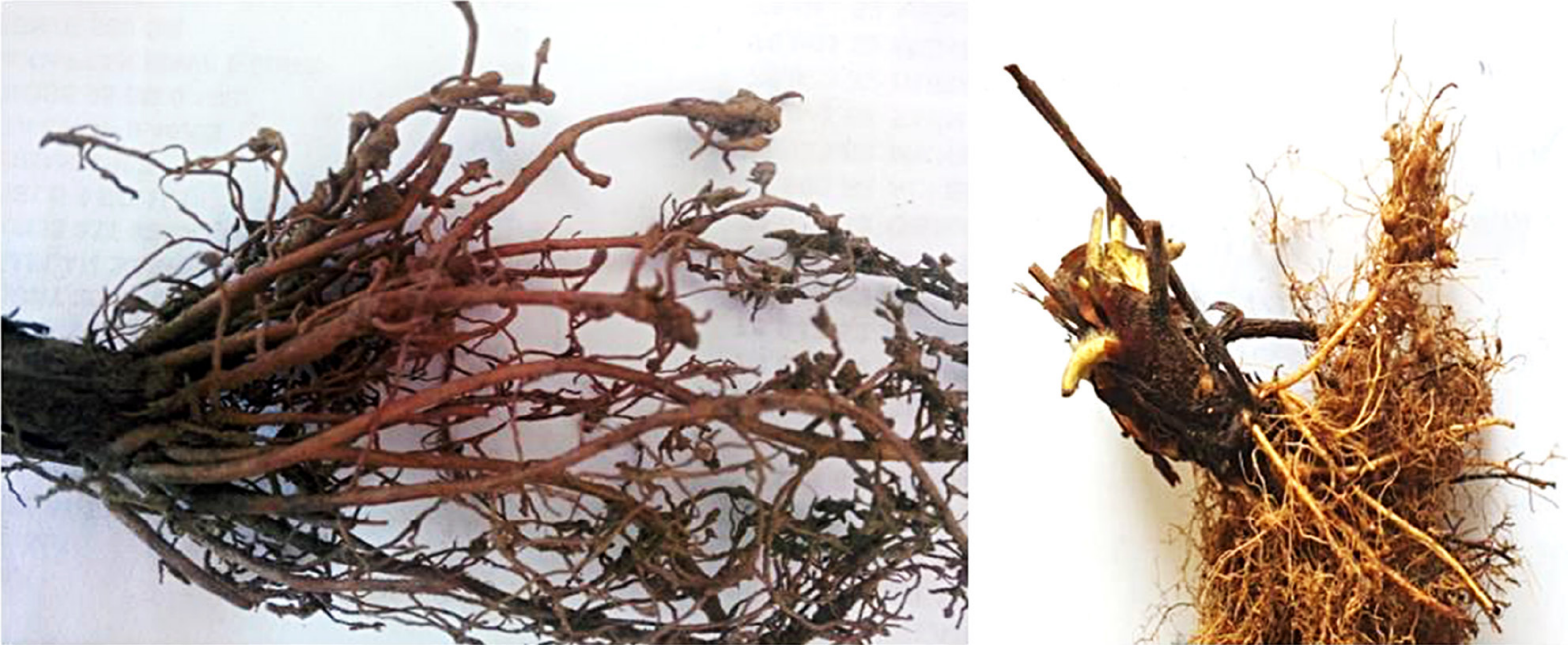
Damage of *Meloidogyne hapla* on strawberry roots.

To determine the presence of *Meloidogyne,* soil and galled plant samples were transferred to Mersin University nematology laboratory (Fig. 1). To isolate nematode individuals, plant roots were cut into small pieces, placed on modified Baermann funnel, and the isolated nematodes were identified under the microscope based on J2 morphological and morphometric parameters (tail length, hyaline terminus length, stylet length, distance between DGO and the stylet base) in Silifke district of Mersin (Hartman and Sasser, 1985). Pathogenicity tests were conducted in the greenhouse at 24 ± 2°C under 16 h/8 h day/night photoperiod with disease-free strawberry plants in pots that contained sandy soil. Two nematode isolates were used in six replicates and inoculation was conducted by releasing 1,000 juveniles per pot. Non-inoculated control plants were irrigated with an equal volume of water. The experiment was terminated on 60th post-inoculation day. Isolation of DNA was conducted on 2 to 3 larvae based on the protocol. The larvae were collected into Eppendorf tubes and WLB (+) was added, and the samples were incubated at 65°C for 1 hr and at 95°C for 10 min to obtain the genomic DNA (Waeyenberge et al., 2000). The species-specific SCAR markers (JMV1, JMV2, and JMVhapla) were used for *M. hapla* (Wishart et al., 2002). The PCR was conducted with the general primers D2\D3 that encode 28S rRNA gene region and PCR products were sequenced.

## Results

### Morphological and molecular identification of the nematode

**Figure 2:**
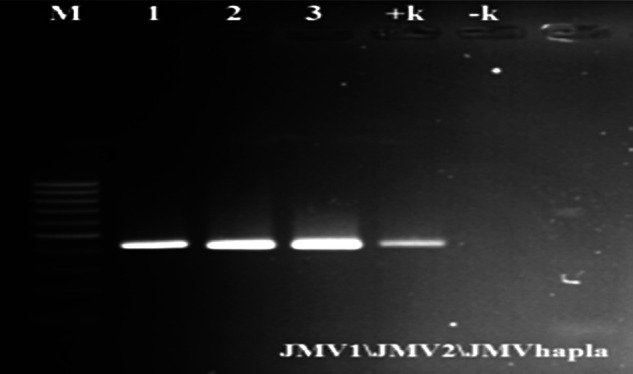
PCR product at 440 bp using species-specific SCAR primers (JMV1, JMV2 and JMVhapla). M: 100 bp molecular size marker, 1,2 and 3: larvae from field infected samples. +k: positive control and –k: negative control.

This study was conducted to determine the species of root-knot nematode that causes galls of various sizes on strawberry roots. The inoculated strawberry plants exhibited typical galling on roots. Koch’s postulates were confirmed by re-isolating the root-knot nematodes in the inoculated plants. The second-stage *M. hapla* juveniles isolated in Silifke demonstrated the following morphometric characters: (*n* = 12), *L* = 371.3 ± 4.52 µm; *a* = 25 ± 1.3; *b* = 4.3 ± 0.2; *c* = 7.6 ± 0.2, *c′* = 4.9 ± 0.3; Stylet = 11.1 ± 0.3 µm; Tail = 49.2 ± 1 µm; hyaline = 13.8 ± 0.6 µm. The species-specific SCAR markers (JMV1, JMV2, and JMVhapla) yielded a 440 bp band specific to *M. hapla* (Fig. 2). The PCR was conducted with the general primers D2\D3 that encode 28S rRNA gene region and PCR products were sequenced. The obtained sequence was 100% identical with *M. hapla*. The gene sequences were uploaded to the GenBank database with access numbers MN897751 and MN895037. Both morphological and molecular diagnostic methods confirmed that the strawberry plants collected in Silifke were infested with *M. hapla*. To our best knowledge, the present study is the first report on plant-parasitic nematode species *M. hapla* infection in strawberries in Turkey. Currently, the adverse effects of RKN on strawberry production in the region is unknown to strawberry farmers.

## Discussion

The species-specific SCAR markers (JMV1, JMV2, and JMVhapla) yielded a 440 bp band specific to *M. hapla*. The present study results were consistent with earlier studies (Adam et al. 2007; Akyazi et al. 2017; Wishart et al., 2002; Zijlstra, 2000). The BLAST analysis demonstrated that the sequences (MN897751 and MN895037) were 100% identical with others (MN475814, MN446015, MK2133550, and MN752204) available in the NCBI database. The present study reported the first identification of *M. hapla* in strawberry fields. In Turkey, it was not identified in strawberry fields in previous studies conducted in Zonguldak and Bartın provinces in the Black Sea Region, Bursa, Yalova, and Istanbul provinces in the Marmara Region, and Mersin province in the Mediterranean Region (Özarslandan, 2019). Several plant-parasitic nematode species were reported in strawberries, among which the most harmful was the northern root-knot nematode (*M. hapla*) (Nyoike et al., 2012). The northern root-knot nematode (*M. hapla*) and root-lesion nematode (*P. penetrans)* are also significant strawberry pests observed in several cultivation regions (Esnard and Zuckerman, 1998; Medina-Minguez et al., 2012; Lopez-Aranda et al., 2016), and were likely introduced to Florida from northern states and Canada via contaminated transplant material (Nyoike et al., 2012). Second-stage *M. hapla* juveniles penetrate the young roots, leading to root galls that disrupt water intake and the physiology of the infected plant, and may lead to severe stunting in sandy soil (Pinkerton and Finn, 2005). *P. penetrans* causes severe root necrosis and could predispose infested roots to secondary infections due to opportunistic fungal pathogens (LaMondia and Martin, 1989). Nematodes lead to significant injuries in strawberry roots, reducing the plant’s water and nutrient intake capacity (Noling, 2016). It was reported that *M. hapla* is common in strawberry fields in other countries. In the present study, *M. hapla* was reported in strawberry cultivation fields for the first time in Turkey. It should be considered that it could spread to fields in other cultivation regions and cause economic losses. In addition to their direct impact, the nematodes could lead to more severe damages in the plant by opening the door to soil-borne pathogens on the wounds they create on the roots. The implementation of integrated control methods in strawberry cultivation fields could increase the yield by reducing nematode damages. Strawberry producers were not aware of the reason for the yield losses since they had no knowledge on nematode damage. Nematode species should be identified in future studies that would be conducted in strawberry cultivation fields in Turkey.
